# Genetic testing of leukodystrophies unraveling extensive heterogeneity in a large cohort and report of five common diseases and 38 novel variants

**DOI:** 10.1038/s41598-021-82778-0

**Published:** 2021-02-05

**Authors:** Nejat Mahdieh, Mahdieh Soveizi, Ali Reza Tavasoli, Ali Rabbani, Mahmoud Reza Ashrafi, Alfried Kohlschütter, Bahareh Rabbani

**Affiliations:** 1 Growth and Development Research Center, Tehran University of Medical Sciences, Tehran, Iran; 2 Rajaie Cardiovascular Medical and Research Center, Iran University of Medical Sciences, Tehran, Iran; 3 Myelin Disorders Clinic, Pediatric Neurology Division, Children’s Medical Center, Pediatrics Center of Excellence, Tehran University of Medical Sciences, Tehran, Iran; 4 Department of Pediatrics, University Medical Center Hamburg Eppendorf, Hamburg, Germany; 5 Iranian Comprehensive Hemophilia Care Center, Tehran, Iran

**Keywords:** Neurodevelopmental disorders, Genetics, Mutation

## Abstract

This study evaluates the genetic spectrum of leukodystrophies and leukoencephalopathies in Iran. 152 children, aged from 1 day to 15 years, were genetically tested for leukodystrophies and leukoencephalopathies based on clinical and neuroradiological findings from 2016 to 2019. Patients with a suggestive specific leukodystrophy, e. g. metachromatic leukodystrophy, Canavan disease, Tay-Sachs disease were tested for mutations in single genes (108; 71%) while patients with less suggestive findings were evaluated by NGS. 108 of 152(71%) had MRI patterns and clinical findings suggestive of a known leukodystrophy. In total, 114(75%) affected individuals had (likely) pathogenic variants which included 38 novel variants. 35 different types of leukodystrophies and genetic leukoencephalopathies were identified. The more common identified disorders included metachromatic leukodystrophy (19 of 152; 13%), Canavan disease (12; 8%), Tay-Sachs disease (11; 7%), megalencephalic leukodystrophy with subcortical cysts (7; 5%), X-linked adrenoleukodystrophy (8; 5%), Pelizaeus–Merzbacher-like disease type 1 (8; 5%), Sandhoff disease (6; 4%), Krabbe disease (5; 3%), and vanishing white matter disease (4; 3%). Whole exome sequencing (WES) revealed 90% leukodystrophies and genetic leukoencephalopathies. The total diagnosis rate was 75%. This unique study presents a national genetic data of leukodystrophies; it may provide clues to the genetic pool of neighboring countries. Patients with clinical and neuroradiological evidence of a genetic leukoencephalopathy should undergo a genetic analysis to reach a definitive diagnosis. This will allow a diagnosis at earlier stages of the disease, reduce the burden of uncertainty and costs, and will provide the basis for genetic counseling and family planning.

## Background

Leukodystrophies and genetic leukoencephalopathies are a large heterogeneous group of genetic diseases affecting the white matter of the central nervous system. The single diseases are rare, but overall they affected 1 per 7663 live births, in a US American study^1^; the estimated prevalence of leukodystrophies is about 1–2/100,000 live births in Germany^2^. Most of these diseases are associated with severe progressive functional losses of motor and cognitive abilities, helplessness and early death. Their causes are either related to primary defects of myelin synthesis and myelin stability, but myelin damage may also be secondary to disturbances outside this structure^3^. Some mitochondrial and lysosomal storage disorders, organic acidemias, other inborn errors of metabolism and vascular disorders are also categorized under genetic leukoencephalopathies^4^.

Leukodystrophies are clinically and genetically heterogeneous disorders; their diagnosis is challenging and nearly half of the patients will remain undiagnosed^5^, putting a high economical and psychological burden on the society and the affected families. Many known genes have been recognized to cause these diseases, though there are many with unknown genetic etiology. Advances in gene sequencing procedures and whole exome sequencing (WES) unravel the genetic causes of leukodystrophies^6^. Genetic testing confirms the diagnosis and may offer a chance for disease-specific palliative treatment or experimental therapies of some diseases (e. g. metachromatic leukodystrophy (MIM 250100), Alexander disease (MIM 203450), and Krabbe disease (MIM 611722)^7,8^. In addition, molecular genetic analysis would help for family screening and reproductive decisions. Most of the pediatric disorders follow an autosomal recessive pattern of inheritance and come from consanguineous marriages which are prevalent in Iran and the Middle East. Despite advances in molecular technologies and the high frequency of genetic diseases in Iran as the crossroads of the Middle East, there is no comprehensive study on genetics of pediatric white matter disorders in this region of the world. The genetic composition of different parts of Iran could be representative of the respective neighbors.

Here, we have evaluated the genetic spectrum of subjects clinically diagnosed with leukodystrophies referred to a tertiary pediatric center in Iran. The purpose of the study was to determine the common types of leukodystrophies and genetic leukoencephalopathies, neurological findings in the patients, and ethnical distribution of the disease.

## Results

### Patients’ demographic data and clinical diagnoses

A total of 152 patients, including 94 (62%) males and 58 females, aged from 1 day to 15 years old, has been clinically diagnosed with leukodystrophy or genetic leukoencephalopathy. The distribution of the more common referred diseases among the patients was as follows: 25 patients clinically diagnosed with MLD^9^, 13 CD, 10 PMLD, 6 PMD, 2 PMD or PMLD, 12 TSD, 10 X-ALD, 8 SHS, 8 MLC, 3 AxD, 3 KD, 4 hypomyelination and congenital cataract (HCC; Hypomyelinating Leukodystrophy 5; HLD5; MIM 253260), 1 Sialic disease, 1 RNAse T2 deficient leukoencephalopathy (MIM 612951), and 2 biotinidase deficiency (MIM 253260).

Totally, 108 of 152 patients (71%) had defined MRI patterns (not available) and were clinically diagnosed with a known leukodystrophy. Measurements of lysosomal enzymes in MLD, KD, TSD and SHS were performed for diagnosis. Urinary sulfatides (for e. g. MLD), plasma very long chain fatty acids (for e. g. X-ALD) were also tested to help the diagnosis. These patients were candidates for single gene analysis.

44 of 152 patients (29%) had no definite MRI pattern and no definite biochemical or single gene test could be performed for them. They were candidates for panel gene analysis and/or WES.

### Demographic, clinical and genetic evaluation of patients confirmed genetically

**Figure 1:**
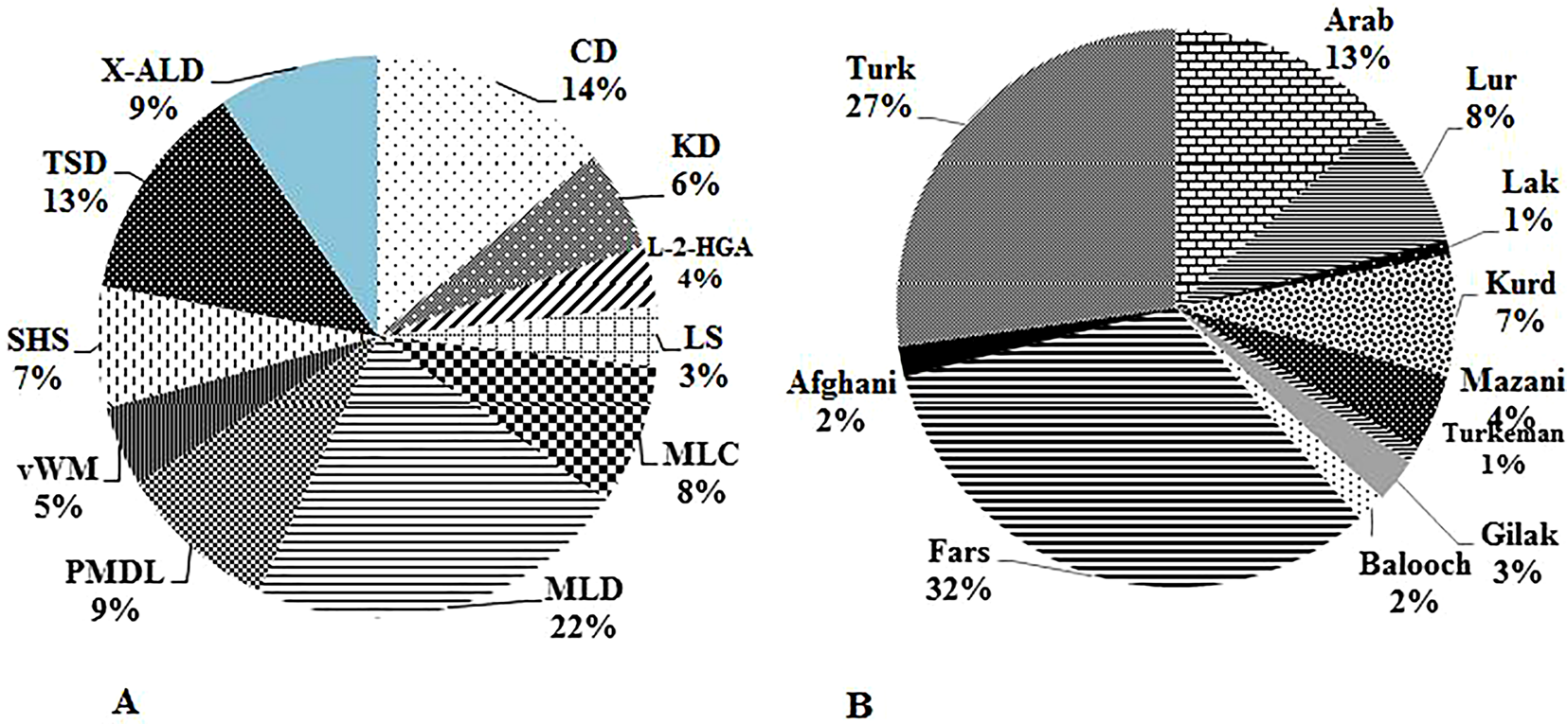
(**A**) The distribution of the most common diagnosed leukodystrophies (86 of 114 patients) in our studied patients include 19 of 86 (22%) MLD, 12 CD, 11 TSD, 8 X-ALD, 8 PMLD1, 7 MLC, 6 SHS, 5 KD, 4 vWM, 3 LS and 3 L-2-HGA. (**B**) The distribution of 114 leukodystrophy genotype positive patients in Iran based on ethnicity.

Thirty-five different leukodystrophies and genetic leukoencephalopathies were identified in this study (Table 1). The clinical characteristics of the most common genetically confirmed patients are summarized in Table 1 and Fig. 1A. The main clinical manifestation was motor regression and neurological complaints including dystonia, hypotonia, developmental delay, ataxia, tremor, seizure, macrocephaly, nystagmus, cognition and learning impairment (Table 1 and Supplementary Table 2).

**Table 1:** The distribution of the leukodystrophies and genetic leukoencephalopathies based on single gene analysis and WES/panel based gene sequencing in 114 positive patients in the studied population.

No	Name of disease	Alternative designation, abbreviation	MIM #	Gene	Location of protein	No of families (%)	Genetic testing		Phenotypes
							Single gene	WES	
**Leukodystrophies**									
1	Metachromatic leukodystrophy	MLD	250100	*ARSA*	ER, Lysosome	19 (16. 7)	16	3	AG:2, MR:15, DD:2, CI:1, speech problem:6
2	Krabbe Disease	KD	245200	*GALC*	Lysosome	5 (4. 4)	3	2	Hypotonia:1, speech problem: 2, Spasticity: 2, AG: 2, Seizure: 2, MR: 5, DD:2
3	Fucosidosis		230000	*FUCA1*	Lysosome	2 (1. 8)	0	2	Hypotonia:1, Dental germination:1, skin lesions:1, AG:1, DD: 2
4	Salla Disease	SD	604369	*SLC17A5*	Lysosomal and cell membrane	1 (0. 9)	1	0	speech problem, Seizure, DD, MD
5	Multiple sulfatase deficiency	MSD	272200	*SUMF1*	ER	1 (0. 9)	0	1	dried skin, spasticity, incapable to walk and talk, R, mental retardation, coarse facial feature
6	RNAse T2 deficiency		612944	*RNASET2*	ER, Lysosome, Extracellular	1 (0. 9)	0	1	Hypotonia, DD
7	X-linked adrenoleukodystrophy	X-ALD	300100	*ABCD1*	Membrane of ER, Mitochondrion, peroxisome and lysosome	8 (7)	8	0	Hypotonia: 2, Vision problem: 1, Feeding problem: 2, AG: 3, Seizure: 2, MR: 2, LD:1, CI: 4
8	Rhizomelic chondrodysplasia punctata	RCDP	601757	*PEX7*	Peroxisome	1 (0. 9)	0	1	coarse facial feature, cataract, digestive problem, DD, MR
9	Zellweger Spectrum	ZS	614883	*PEX13*	Peroxisome membrane	1 (0. 9)	0	1	Hypotonia, Seizure, MR, feeding problem
10	D-bifunctional protein deficiency	DBPD	601860	*HSD17B4*	Peroxisome	1 (0. 9)	0	1	swallowing problem, walking difficulty, speech problem, MR
11	Canavan Disease	CD	271900	*ASPA*	Nucleus, Cytoplasm	12 (10. 5)	12	0	Hypotonia:8, Nystagmus and eye problem:5, Macrocephaly:9, Spasticity:3, Irritable:6, Seizure:3, R: 9, DD:7
12	Pelizaeus–Merzbacher-like disease type	PMLD	260600	*GJC2*	Cell membrane, gap junction	8 (7)	6	2	Hypotonia: 6,Nystagmus: 8, Ataxia: 4, Speech problem:6, DD:6
13	Megalencephalic leukoencephalopathy with subcortical cysts	MLC	604004	*MLC1*	ER and cell membrane	7 (6. 1)	7	0	Macrocephaly:7, Dystonia: 2, AG: 4, Seizure: 2, MD:2, MR:5
14	Vanishing white matter disease	vWM	606273 603945 606687	*EIF2B3* *EIF2B5* *EIF2B4*	Cytosol Cytosol, nucleus Cytosol	1 (0. 9) 2 (1. 8) 1 (0. 9)	0 0 0	1 2 1	MR:4, Hypotonia: 3,Tremor: 2, AG: 2, Seizure: 2, speech problem 1
15	Hypomyelination-hypogonadotropic hypogonadism-hypodontia	4H	614366, 614381	*POLR3A* *POLR3B*	Nucleus Nucleus	1 (0. 9) 1 (0. 9)	0 0	1 1	Hypotonia: 2, speech problem: 2, Tremor:1, ataxia:2, AG: 2, Seizure:2, MR:1, DD:1, nystagmus:1
16	hypomyelination and congenital cataract	HCC	610532	*FAM126A*	Cytosol	1 (0. 9)	0	1	congenital cataract
17	Pelizaeus–Merzbacher disease	PMD	312080	*PLP1*	Cell (myelin) membrane	1 (0. 9)	1	0	MR, Hypotonia, nystagmus
18	Alexander disease	AxD	203450	*GFAP*	Cytoplasm	1 (0. 9)	1	0	Seizure, R, DD, hypotonia
19	infantile neuroaxonal dystrophy/atypical neuroaxonal dystrophy	INAD	603604	*PLA2G6*	Peripheral membrane	1 (0. 9)	0	1	Hypotonia, bristling head, Seizure
20	Hypomyelinating leukodystrophy-9	HLD9	616140	*RARS*	Cytosol	1 (0. 9)	0	1	Spasticity, hypotonia, MD
**Genetic Leukoencephalopathies**									
21	Tay-Sachs Disease	TSD	272800	*HEXA*	Lysosome	11 (9. 6)	11	0	Vision problem and nystagmus:8, R:6, DD:4
22	Sandhoff disease	SHS	606873	*HEXB*	Lysosome	6 (5. 3)	5	1	Visual problem:2, Seizure:1, R:4, DD:4
23	Gangliosidosis	GM1	230500	*GLB1*	Lysosome	1 (0. 9)	0	1	
24	Neuronal Ceroid-Lipofuscinoses	NCL	204300	*PPT1,* *CLN6*	Extracellular, Lysosome ER membrane	1 (0. 9) 1 (0. 9)	0 0	1 1	Hypotonia:1, speech problem:2, AG: 2, Seizure:2, MR:2, DD:1
25	Mucopolysaccharidosis type IIIB	MPS IIIB	609701	*NAGLU*	Lysosome	1 (0. 9)	0	1	coarse facial feature, macrocephaly
26	Cockayne Syndrome	CS	609413	*ERCC6*	Nucleus	1 (0. 9)	0	1	Microcephaly, AG, MR/R
27	Biotinidase deficiency	BTD	253260	*BTD*	Extracellular	1 (0. 9)	1	0	Seizure
28	L-2-hydroxyglutaric aciduria	L-2-HGA	236792	*L2HGDH*	Mitochondrion	3 (2. 6)	2	1	Hypotonia:1, Macrocephaly:1, speech problem: 1, tremor: 1, AG: 1, Seizure: 1, DD: 3, LD:1, Mental retardation:3
29	Glutaric acidemia IIC	GAIIC	231680	*ETFDH*	Mitochondrion inner membrane	1 (0. 9)	0	1	Walking problem, speech problem, digestive problem, MR
30	Mitochondrial DNA depletion syndrome 5	MDDS5	612073	*SUCLA2*	Mitochondrion	1 (0. 9)	0	1	Dystonia, R, DD
31	Ataxia neuropathy spectrum	ANS	203700	*POLG*	Mitochondrion	2 (1. 8)	0	2	Speech difficulty:1, walking difficulty:1, vision problem:1, ataxia:1, Seizure:1, DD:2
32	Leigh syndrome	LS	185620	*SURF1*	Mitochondrion inner membrane	3 (2. 6)	0	3	Muscle weakness:3, walking problem:3, swallowing problem:2, R:3, DD:1
33	Mitochondrial complex I deficiency, nuclear type 5	MC1DN5	618226	*NDUFS1*	Mitochondrion inner membrane	1 (0. 9)	0	1	Walking problem, Seizure, MR
34	Mitochondrial complex I deficiency, nuclear type 3	MC1DN3	618224	*NDUFS7*	Mitochondrion	1 (0. 9)	0	1	Hypotonia, Seizure
35	succinate dehydrogenase complex assembly factor 1 deficiency	MCIID	252011	*SDHAF1*	Mitochondrion	1 (0. 9)	0	1	Speech problem, walking problem, R
Total						114 (75)	74 (49%)	40 (26%)	

DD: Developmental delay; LD: learning difficulties; CI: Cognitive impairment; MR/R: Motor regression/retardation; MD: motor delay, MD; AG: Abnormal gait; ER: Endoplasmic reticulum.

114 (75%) patients were confirmed based on genetic testing. Male consist of 73 of 114 (64%) of patients. The mean age of onset was 5yrs and 1 m ± 18yrs and 11 m. 94 of 114 (82. 5%) cases were born in a consanguineous family. The ethnicity of these patients is compared in Fig. 1B. The ethnical distribution showed higher incidence in Fars 32%; other ethnical distribution included 27% in Turk, Arab 13%, Lur 8%, Kurd 7%, Mazani 4%, Gilak 3%, and the rest Balooch, Afghan, Lak, and Turkeman (Fig. 1B). Based on age of onset of disease, 47 infantile (41%, I), 17 late infantile (15%, LI), 29 early juvenile (25%, EJ), 19 late juvenile (17%, LJ) and 2 adults (A) were available (Supplementary Table 2).

**Figure 2:**
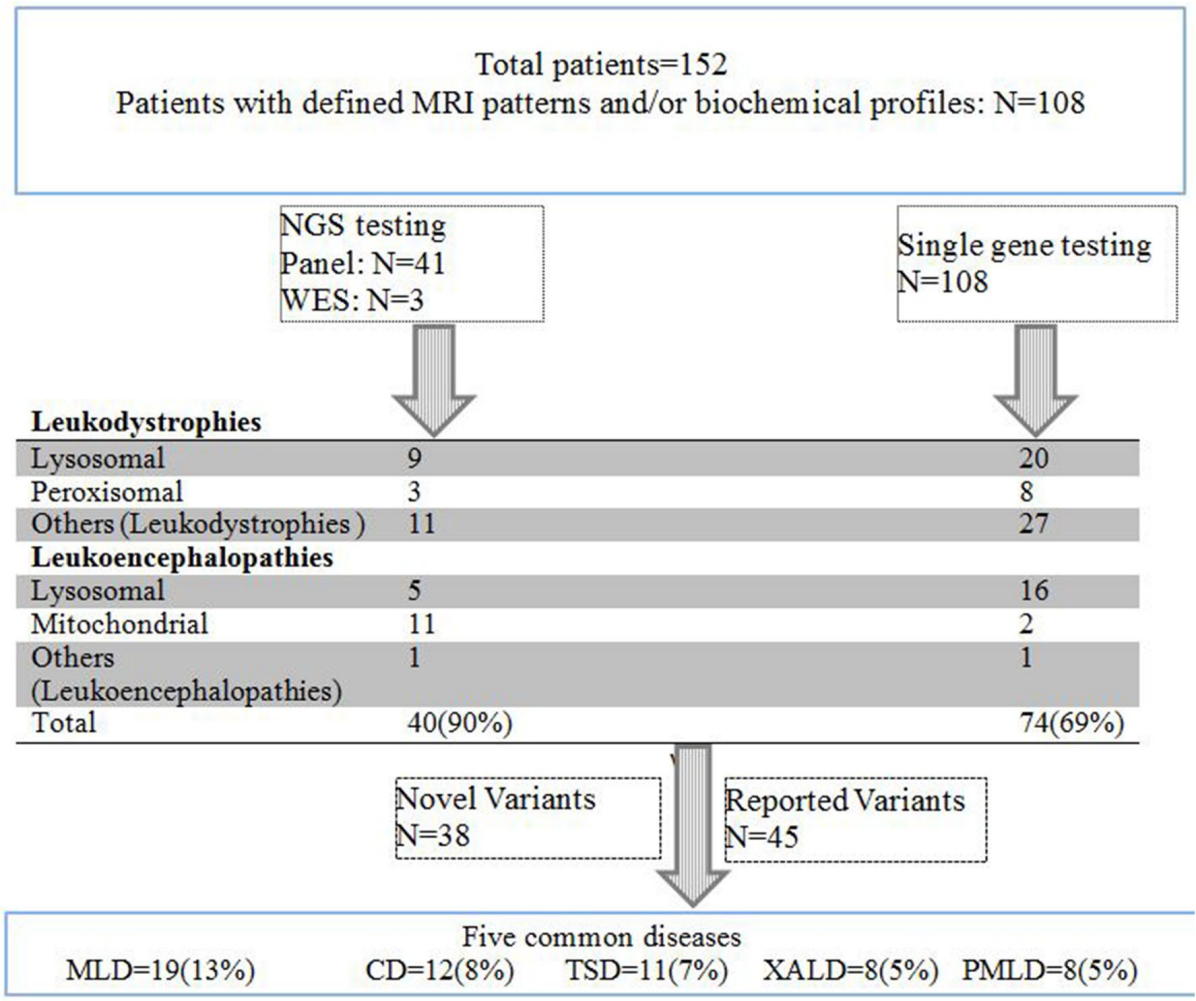
The flow chart of patients undergoing different genetic testing in this cohort and identified leukodystrophies. Fifty of 114 patients were diagnosed as lysosomal disorders (29 lysosomal LD and 21 lysosomal gLE). Forty-one patients genetically were confirmed for MLD, TSD, SHS and KD. Eleven patients were diagnosed as peroxisomal disorders which eight of them were X-ALD. One patient had peroxisomal single enzyme beta oxidation deficiency, and two patients had peroxisomal biogenesis disorders. Forty patients diagnosed as errors of intermediary metabolism, consisted of 12 CD, 8 PMLD and 7 MLC (Table 1). CD as the most common degenerative cerebral diseases, due to abnormal amino acid/organic acid metabolism, accounted for the second most common disease in our population. PMD and PMLD are disorders of myelin genes. 4 patients had vWM, 2 patients with hypomyelination-hypogonadotropic-hypogonadism-hypodontia, 1 hypomyelination and congenital contract, 1 PMD, 1 AxD, 1 infantile neuroaxonal dystrophy/atypical neuroaxonal dystrophy, 1 hypomyelination leukodystrophy 9 (HLD9, MIM 616140), 1 Cockayne syndrome CS, MIM 133540), and 1 biotinidase deficiency. Thirteen patients diagnosed with mitochondrial genetic leukoencephalopathies; Leigh syndrome and L-2-HGA accounted for 4 and 3 of them, respectively.

38 of 152 (25%) patients were not genetically confirmed based on genetic analysis. Some candidates of single gene analysis were not tested for panel based analysis because the parents were not satisfied for the test performance (Fig. 2). In addition, panel negative patients did not perform WES.

### Single gene analyses

Sixteen patients had mutations in the *ARSA* gene (MLD), 8 in *ABCD1* gene (X-ALD), 12 in *ASPA* gene (CD), 3 in *GALC* (GLB), 7 in *MLC1* gene (MLC), 1 in *GFAP* gene, 1 in *PLP1* gene (PMD), 6 in *GJC2* gene (PMLD), 11 in *HEXA*, 5 in *HEXB,* 2 in *L2HGDH*, 1 in *BTD* and 1 in *SCL17A5* gene (Table 1). Totally, 74 out of 108 (69%) patients were genetically diagnosed based on single gene analysis (Fig. 2).

### Next generation sequencing: gene-panel and WES

Gene-panel and WES identified 40 of 44 (90%) patients having leukodystrophies and leukoencephalopathies (Table 1, Supplementary Table 2). Four cases did not show any variants with multigene panel analysis of leukodystrophies (Fig. 2).

### Frequency of lysosomal, peroxisomal, mitochondrial and errors of intermediary metabolism

Fifty of 114 patients were diagnosed as lysosomal disorders (29 lysosomal LD and 21 lysosomal gLE) (Table 1, Fig. 2 and Supplementary Table 2).

Eleven patients were diagnosed as peroxisomal disorders which eight of them were X-ALD (Table 1, Fig. 2 and Supplementary Table 2).

Forty patients diagnosed as errors of intermediary metabolism, consisted of 12 CD, 8 PMLD and 7 MLC (Table 1). CD as the most common degenerative cerebral diseases, due to abnormal amino acid/organic acid metabolism, accounted for the second most common disease in our population (Table 1, Fig. 2 and Supplementary Table 2).

Thirteen patients diagnosed with mitochondrial genetic leukoencephalopathies (Table 1, Fig. 2 and Supplementary Table 2).

### Common variants

Five common diagnosed leukodystrophies accounted for 51% (58 of 114 patients) in our studied patients included 19 of 58 (33%) MLD, 12 CD, 11 TSD, 8 X-ALD and 8 PMLD (Fig. 2).

Six common mutations were found including p.Gly311Ser (in 6 MLD patients), c.465 + 1G > A (in 3 MLD patients), c.634 + 1G > T (in 6 CD patients), c.237_238insA (in two homozygous and one compound heterozygous CD patients), c.1528C > T (in 4 TSD patients) and c.449_455delTCCTGCT (two homozygous and one heterozygous MLC patients).

### Novel variants

Thirty-eight novel variants were identified in 40 patients (Table 2). Each of *ABCD1* and *GJC2* showed four novel variants. Following genes had each two novel variants: *ASPA, FUCA, GALC, HEXA, L2HGDH* and *MLC1* (Table 2). The variants were classified according to ACMG guideline; 11 variants met the criteria for being pathogenic, 17 and 10 variants were likely pathogenic and VUS, respectively.

**Table 2:** Novel variants identified in this study.

No	Nucleotide change	AA change	Gene	no. of patients	Zygosity	ACMG	MutationTaster	Polyphen-2	CADD
1	c. 2099A > C	p. Asn700Thr	*POLR3B*	1	Hom	Likely pathogenic (2)	DC	PD 0. 998	27. 2
2	c. 786A > C	p. Gln262Asp	*SLC17A5*	1	Hom	Likely pathogenic (2)	DC	PD 1. 000	24. 2
3	c. 904_905delinsAT	p. Glu302Met	*ABCD1*	1	Hemi	Likely pathogenic (2)	DC	NA	26. 8
4	c. 1628C > G	p. Pro543Arg	*ABCD1*	1	Hemi	Likely pathogenic (2)	DC	PD 1. 000	23. 8
5	c. 2002A > G + c. 1021G > T	p. Thr668Ala + p. Ala341Ser	*ABCD1*	1	Hemi	Likely pathogenic (2) Likely pathogenic (2)	DC	PD 0. 761	23. 7
6	c. 839G > C	p. Arg280Pro	*ABCD1*	1	Hemi	Likely pathogenic (2)	DC	PD 1. 000	32
7	c. 233C > A	p. Ser78Ter	*RNASET2*	1	Hom	Pathogenic (1)	DC	NA	36
8	c. 437_449delCTCTGGCTCCACT	p. Ser146TyrfsX7	*ASPA*	1	Hom	Pathogenic (1)	DC	NA	34
9	c. 359C > T	p. Ser120Phe	*ASPA*	1	Hom	Uncertain significance (3)	DC	PD 1. 000	29. 1
10	c. 866G > A	p. ser289Ile	*EIF2B4*	1	Hom	Uncertain significance (3)	DC	B 0. 002	22. 9
11	c. 422G > T	p. Gly141Val	*FUCA1*	1	Hom	Likely pathogenic (2)	DC	PD 1. 000	28. 8
12	c. 82delG	p. Val28CysfsX105	*FUCA1*	1	Hom	Pathogenic (1)	DC	NA	16. 62
13	c. 830G > A	p. Ser277Asn	*GALC*	1	Hom	Likely pathogenic (2)	DC	PD 0. 946	23. 9
14	c. 1942A > T	p. Lys648Ter	*GALC*	1	Hom	Uncertain significance (3)	DC	NA	36
15	c. 408 + 1G > C	–	*L2HGDH*	1	Hom	Pathogenic (1)	DC	NA	34
16	c. 1213A > G	p. Arg405Gly	*L2HGDH*	1	Hom	Uncertain significance (3)	DC	PD 1. 000	22. 7
17	c. 183C > A	p. Cys61Ter	*MLC1*	1	Hom	Pathogenic (1)	DC	NA	37
18	c. 819C > G	p. Phe273Leu	*MLC1*	1	Hom	Uncertain significance (3)	DC	PD 0. 990	24. 1
19	c. 571_572insC	p. Thr195AspfsX69	*GJC2*	1	Hom	Pathogenic (1)	DC	NA	17. 5
20	c. 118G > C	p. Ala40Pro	*GJC2*	2	Hom	Likely pathogenic (2)	DC	PD 1. 000	24. 6
21	c. 733 T > A	p. Cys245Ser	*GJC2*	2	Hom	Likely pathogenic (2)	DC	PD 1. 000	25. 1
22	c. 883C > T	p. Gln295Ter	*GJC2*	1	Hom	Likely pathogenic (2)	DC	NA	38
23	c. 529_531delAAA	p. Lys177del	*PEX13*	1	Hom	Pathogenic (1)	DC	NA	22. 2
24	c. 345C > G	p. Ile115Met	*PEX14*	1	Het	Uncertain significance (3)	DC	PD 0. 999	23. 5
25	c. 655_657delATT	p. Ile219del	*HEXB*	1	Hom	Pathogenic (1)	DC	NA	20. 3
26	c. 754C > T	p. Arg252Cys	*HEXA*	1	Hom	Likely pathogenic (2)	DC	PD 1. 000	30
27	c. 1147-1G > T	–	*HEXA*	1	Hom	Pathogenic (1)	DC	NA	28. 3
28	c. 16C > T	p. Arg6Cys	*PLA2G6*	1	Hom	Uncertain significance (3)	DC	PD 0. 994	25
29	c. 416 T > A	p. Leu139Gln	*GLB1*	1	Hom	Likely pathogenic (2)	DC	PD 1. 000	29. 3
30	c. 997G > T	p. Asp333Tyr	*SUCLA2*	1	Hom	Likely pathogenic (2)	DC	PD 1. 000	31
31	c. 3482 + 6C > T	–	*POLG*	1	Hom	Uncertain significance (3)	DC	NA	9. 6
32	c. 29A > C	p. Gln10Pro	*SDHAF1*	1	Hom	Uncertain significance (3)	DC	PD 1. 000	27
33	c. 808_812delGAGCA	p. Glu270SerfsX20	*SURF1*	1	Hom	Pathogenic (1)	DC	NA	35
34	c. 362 + 5G > A	-	*PPT1*	1	Hom	Pathogenic (1)	DC	NA	21. 9
35	c. 659A > C	p. Tyr220Ser	*CLN6*	1	hom	Uncertain significance (3)	DC	PD 0. 986	32
36	c. 392C > A	p. Thr131Lys	*HSD17B4*	1	Hom	Likely pathogenic (2)	DC	PD 0. 985	33
37	c. 1285G > A	p. Val429Met	*NDUFS1*	1	Hom	Likely pathogenic (2)	DC	PD 0. 971	28. 8
38	c. 415G > A	p. Asp139Asn	*NDUFS7*	1	Hom	Likely pathogenic (2)	DC	PD 1. 000	25. 5

DC: disease causing, PD: probably damaging, Hom = homozygous, B = benign, NA = not available.

## Discussion

Genetic diagnosis of childhood leukodystrophies is rapidly increasing throughout the past years in Iran and worldwide; approximately, 30 leukodystrophies and more that 60 disorders have been classified as genetic leukoencephalopathies^4^. This study provides a comprehensive spectrum of leukodystrophies and other genetic leukoencephalopathies in Iran as referred to a tertiary pediatric center. Totally, 35 types of leukodystrophies were determined in the studied population. Based on pattern of brain MRI and single gene analysis, approximately 69% (74 of 108) of the referred patients were confirmed by direct Sanger sequencing. Clinical diagnosis reduced the number of genes to be evaluated. Panel based analysis also confirmed leukodystrophies in 90% (40 of 44) of the cases. Our diagnostic rate of panel-based analysis was comparable to other studies^6^. Four patients were genetically undiagnosed with panel-based/WES studies and WGS is needed to define the causes. Consequently, we had 25% (38 of 152) unsolved genetic cases and the diagnostic rate was 75% (114 of 152) of leukodystrophies and genetic leukoencephalopathies in the study. Various novel variants identified, show that a high rate of allelic heterogeneity exists among our patients. A specific composition of population living in Iran complicates this picture; different ethnicities with specific cultural customs demand to run more specific investigations on each population.

MLD was the most common cause of leukodystrophies in our population^9^. The next diseases were CD, TSD, PMLD, X-ALD and then MLC. MLC is the most common (6 of 23) among Turk patients while PMLD may be common among Arab population in our study. Moreover, ten common diseases of this study, compromise 70% of all recognized patients (80 of 114) (Table 1). A recent study showed that peroxisomal disorders are identified to be common. Although other common disorders including Aicardi Goutières Syndrome, *TUBB4A*-related leukodystrophy, *POLR3*-related Leukodystrophy and Pelizaeus–Merzbacher Disease were not found in our study with a high frequency^10^. *ABCD1* had the highest relative frequency in their study while *ARSA* was the most common in our population.

Clinically, we had unsolved cases due to variable phenotypic features or overlapping neurological manifestations which were candidates of gene-panel and/or WES analysis. Despite we had patients with no genetic diagnosis even though they had undergone panel-based analysis. This could be due to intronic variants, copy number variations, unknown gene defects, and multigenic effect. Therefore, more genetic analysis should be performed for these cases and they could benefit from reanalysis of exome sequencing data, genome sequencing and transcriptomics. For rare diseases genetic analysis, NGS may unravel more genes relating to leukodystrophies in patients with unsolved genetics^6,11^.

Lysosomal diseases had 43% incidence in our studied population which could be managed at earlier age of diagnosis. Individuals with known causal variants benefit from unexpected clinical presentations, prognosis, palliative treatment and avoiding unnecessary treatments. Hematopoietic stem cell transplantation (HSCT) has been used for lysosomal storage diseases^7^. Some of our patients might potentially have benefitted from HSCT at early stages of the disease. However, patients’ follow up for HSCT is out of the scope of this study.

Some have an ethnic-specific distribution, e. g. TSD in Ashkenazi Jewish population, GM1 gangliosidosis in Rudari isolate and MLD in Western Navajo Nation^12^. MLD patients were from western part of Iran^9^. Four of our TSD patients were from northern parts of Iran.

The peroxisomal disorders, as a heterogeneous group, occur due to a defect in function (e. g. X-ALD) and biogenesis (e. g. Zellweger spectrum) of peroxisomes. X-ALD is the most common peroxisomal disorder caused by mutation in the *ABCD1* gene co-expressed with *HSD17B4* gene. Patients with X-ALD could benefit from HSCT^13^ or hematopoietic stem-cell gene therapy^8^.

CD is the second frequent disease in our study. It is the most common disease during infancy and has been observed mainly in Ashkenazi Jews while in our study patients were from various ethnicities. Various experimental therapies for Canavan patients are under investigation^14^. Patients with known genetic etiology may benefit from such experimental therapies.

PMLD is responsible for 8% of hypomyelinating leukodystrophy patients^15^. In this study 7% of the patients had the disease. In addition to *GJC2*, mutations in other genes such as a Myelin-associated glycoprotein (*MAG*) gene have been reported to cause PMLD^16^. *GJC2* is co-expressed with *PLP1* and interacts with products of *FAM1256A*, *POLR3A* and *EIF2B5* genes. Our results highlighted that PMLD may have a higher frequency than PMD in our population especially in Arab and Fars ethnicities. Also, six of MLC patients were from Turk ethnicity; it may be a common disorder and limit to specific ethnicity e. g. from Turkey.

11% of patients diagnosed with mitochondrial genetic leukoencephalopathies; Leigh syndrome and L-2-HGA accounted for 4 and 3 of them, respectively. Leigh spectrum was due to *SURF1*. Also, it was due to *NDUFS1, NDUFS7* and *SDHAF1* genes. *L2HGDH* encoding mitochondrial L-2-hydroxyglutarate dehydrogenase may be common in our ethnicities. The mechanism of leukodystrophy is very complicated and there may be proteins involved in disease progress which show overlapping phenotype but have no or unknown interaction with each other.

### Analysis of founder effect and hotspot mutations

Ancestral or founder effect or a genetic signature within an ethnicity usually leads to a high frequency and homozygosity of a mutation in that cohort; in contrast, if a specific mutation is distributed uniformly among many ethnicities, it is known as a mutational hotspot. Haplotype analysis is used to define recognized that a mutation is a hotspot or a founder one. The studied mutations of *ABCD1* (c. 1415_1416delAG), *ASPA*(c. 634 + 1G > T and c. 237_238insA) and *HEXA* (c. 1528C > T) show a wide distribution around the world^17–20^; especially c. 634 + 1G > T in *ASPA* gene has been reported from Turkey for the first time and we found it in patients from Fars, Afghani, Lur and Arab ethnicities^18^. These mutations are considered as hotspots i.e. they are mutated in many populations. Contrarily, mutations of *MLC1* (c. 177 + 1G > T and c. 449_455delTCCTGCT) may have ancestors in Turk population. Especially, the c. 449_455delTCCTGCT variant was observed in three families; it may be originated from a founder ancestor in Turk population and it previously has been reported from Turkey^21^.

### Challenges and limitations

We have not included all the affected patients in our registry, only the patients referred to our center for genetic testing were accounted in this study. In addition, Children’s Hospital is a tertiary center in Tehran and some patients around the country may have not been registered and/or died previously before registration. Therefore, a multicenter registry is needed. The incidence of the disease in this part of the world may be different due to consanguineous marriages. Ethnical background had higher incidence in Fars and Turk; however, the population of these ethnicities is also high in Iran.

## Conclusion

In conclusion, five common disorders are responsible for more than fifty percent of leukodystrophies in this region. Considering Iran as the crossroad of the Middle East is composed of more than 15 ethnicities^22^, it may reflect the distribution of leukodystrophies in the Middle East especially its neighboring populations. For instance, PMDL may be common among Arab countries while MLC may have a high frequency in Turkish countries. Genetic analysis provides diagnostic confirmation of the disease, and physicians are allowed for prognosis and management of patients and affected families. Genetic testing following counseling decreases further worry of the family about the diagnosis and further costs. The mortality rate in affected families is very high and it underscores the necessity of genetic testing in the country. Moreover, this study provides information to help for future therapeutic planning’s in the country. This will allow a diagnosis at earlier stages of the disease, reduce the burden of uncertainty and costs, and will provide the basis for genetic counseling and family planning.

## Methods

### Patients

Clinically diagnosed patients with white matter deterioration were enrolled in the study from different ethnicity of Iran between 2016 and 2019. Clinical characteristics of leukodystrophies and leukoencephalopathies were approved by pediatric neurologists. Demographic data, medical and family history, physical evaluations, neurological examinations, magnetic resonance imaging (MRI), and laboratory testing of each patient were recorded for each patient. The study was approved by the ethical committee of Iran University of Medical Sciences. Informed consent was obtained for genetic testing from the parents of patients. All experimental protocols were approved by Growth and Development Research Center, Tehran University of Medical Sciences and performed in accordance with relevant guidelines and regulations.

### Study strategy

#### Single gene analysis based on clinical diagnosis

Patients with a strongly suspected cause of their leukodystrophy were genetically analyzed for the respective relevant gene. These studies included the genes of metachromatic leukodystrophy (MLD), Canavan disease (CD, MIM 271900), X-linked adrenoleukodystrophy (X-ALD, MIM 300100), Alexander disease (AxD), Tay-Sachs disease (TSD, MIM 272800), Sandhoff disease (SHS, MIM 268800), Krabbe disease (KD), megalencephalic leukodystrophy with subcortical cysts (MLC, MIM 604004), Sialic acid storage disease (SD, MIM 269920), Pelizaeus–Merzbacher disease (PMD, MIM 312080), and Pelizaeus–Merzbacher-like disease type 1 (PMLD1, MIM 608804).

DNA was extracted and amplified by using specific designed primers for coding regions (exons and exon–intron boundaries). The selected genes associated with leukodystrophy were classified to inherited autosomal recessive diseases: *ARSA*(NM_000487. 5; 605908 )*, GALC*(NM_000153. 3; 606890), *MLC1*(NM_015166. 3; 605908), *BTD*(NM_000060. 4; ), *GFAP*(NM_002055. 4; 137780), *GJC2*(NM_020435. 3; 608803), *HEXB*(NM_000521. 3; MIM 606873), *HEXA*(NM_000520. 5; MIM 606869)*, ASPA*(NM_000049. 2; 608034) and *SLC17A5*(NM_012434. 5 MIM, 604322), *FAM126A*(NM_032581. 3; 610531)*,* and X-linked recessive *ABCD1*(NM_000033. 3; 300371), and *PLP1*(NM_001128834. 2; 300401), respectively. Direct sequencing was performed by BigDye termination method ABI 3500 (Applied BioSystems, US).

#### Next generation sequencing: gene-panel and whole exome sequencing (WES)

Those patients (n = 41) with indefinite clinical diagnosis or overlapping symptoms and neurological findings underwent panel gene analysis to detect the genetic cause. Panel based gene analysis was performed for cases for 59 genes involving in leukodystrophy, leukoencephalopathy and vanishing matter white disease (Supplementary Table 1). The coding regions and exon–intron boundaries of the genes were enriched using NimbleGen kit (NimbleGen, Roche, Basel, Switzerland). Sequencing analysis was performed by Illumina, Hiseq2000 (Illumina, San Diego, California, USA). Reads were aligned using Burrows–Wheeler Aligner (BWA) on reference genome (hg19), called by SAMTools and annotated by GATK and ANNOVAR. Based on, 1000Genome and dbSNP database variant were selected for analysis. Coverage of target region with at least depth of 30X was 99%. In addition, WES was only performed with an average coverage depth of ≈100X for three patients. Sanger sequencing was done for the candidate variants in the affected families.

#### Variant categories

The sequence data were compared with public databases and filtered to find out the candidate variants according to published pipelines. The candidate variants were categorized as the previously reported pathogenic variants and novel variants. ACMG guideline criteria were used to interpret novel variants and classify them^23^.

### In silico analyses

#### Pathogenic effect

According to HGVS (http://varnomen.hgvs.org/.hgvs.org/), novel variants were named as missense, nonsense, splice site, intronic, regulatory and indel. The following software tools were applied to predict the pathogenic effects of novel variants: polymorphism phenotyping (PolyPhen-2v2.1)^24^, combined annotation dependent depletion (CADD)^25^ and MutationTaster^26^.

### Ethics approval and patients’ consent

Ethical approval was supported by Growth and development research center, Tehran University of Medical Sciences ID number 98–02-80–43,432 and Iran University of Medical Sciences (IR.IUMS.REC.1399.817). Informed consent was obtained from the parents of patients.

### Consent for publication

All contributing authors have read the manuscript and given their consent for the publication of this study.

## Supplementary Information

Supplementary Information.

## Data Availability

There are no additional unpublished data.
