# Supplementary material for: Genetic testing of leukodystrophies unraveling extensive heterogeneity in a large cohort and report of five common diseases and 38 novel variants
Source: Sci Rep. 2021 Feb 5;11:3231. doi: 10.1038/s41598-021-82778-0 (PMC7864965; doi:10.1038/s41598-021-82778-0)
Supplement: Supplementary file 1 — Supplementary Information. [file 41598_2021_82778_MOESM1_ESM.docx]

Supplementary

**Genetic testing of leukodystrophies unraveling extensive heterogeneity in a large cohort and report of five common diseases and 38 novel variants**

Nejat Mahdieh^1,2^, Mahdieh Soveizi^2^, Ali Reza Tavasoli^3^, Ali Rabbani^1^, Mahmoudreza Ashrafi^3^, Alfried Kohlschütter^4^, Bahareh Rabbani^1,5^ *

1. Growth and development research center, Tehran University of Medical Sciences, Tehran, Iran
2. Rajaie Cardiovascular Medical and Research Center, Iran University of Medical Sciences, Tehran, Iran
3. Myelin Disorders Clinic, Pediatric Neurology Division, Children's Medical Center, Pediatrics Center of Excellence, Tehran University of Medical Sciences, Tehran, Iran
4. University Medical Center Hamburg Eppendorf, Department of Pediatrics, Hamburg, Germany
5. Iranian comprehensive Hemophilia care center, Tehran, Iran

*Correspondence to: Bahareh Rabbani, PhD, Assistant Professor

Growth and development research center, Tehran University of Medical Sciences, Tehran, Iran

Children Medical Hospital Center, Gharib St, Blv. Keshavarz, Tehran Iran

[baharehrabbani@yahoo.com](mailto:baharehrabbani@yahoo.com)

Tel/fax: +982166575730

Supplementary Table 1: primers for amplifying coding regions of the studied genes

| Gene | Forward Primer (5’ to 3’) | Reverse Primer (5’ to 3’) |
| --- | --- | --- |
| ***ARSA*** |  |  |
| Exon 2 | CTGACCATCTGAGGGAGTTAG | TACGACTGCATGTACGGAC |
| Exon 3 | GTCCTATTCATGACCAGCCAC | ACTGCCTCTGTGTTCTGGTAGC |
| Exon 4 | AGGTATTATTGACTCTGTTGAAGC | CATGGTGAAACTCCGTCTGTAC |
| Exon 5 | CCAGTCTTTATTCACTATGCATC | TTTCTTGAATGTGTCTATCCTGG |
| Exon 6 | TTGTTGCCCAGGTTGGAGT | TGTACGCGAAGTGCTGTATG |
| Exon 7 | TCAATGCCTCGCTCAAGTATC | AGACTTTCTAGACACAGGGTTGC |
| ***GALC*** |  |  |
| Exon1 | ACAGACACGCGAGCGATAAGA | CTGCCTGTCTGGTTCGTGTTAAA |
| Exon2-3 | GTGCGTGAACACTGTAGACAGATAT | ACACATATTCTGAAATCACAGTCCA |
| Exon 4 | GAGTGAGATGGTCCTAGGAAGTAC | TTAATAGAGATTCCACCAACACG |
| Exon 5 | AATAGCGCCAGCACCGTTATAC | CCTCATGGCATAAAATGGTTAGTCA |
| Exon 6 | GATGAGAGAAATGGTATCGTAACG | GTCACAACCAGCAAAATAAATAACT |
| Exon 7 | CCAGAACGCTGATTTGTAAAGATA | CATGCATGCTTCAGGTAAGTG |
| Exon 8 | TATATAAGTGCCACGTCGTGACC | CAATCATTGAATCTAGGCTGGAAG |
| Exon 9 | TATTACAATGTACCCCACACTGGA | CTTAAAACACGCATAGACACACAG |
| Exon 10 | CATCATTCCAGGAATCTTCTTGC | TGCTATGTTTTACGACTCATGGC |
| Exon 11 | TCTTGGGCATTAACTGTTGAAC | CTCTGTCAATTCATATGCAAACTG |
| Exon 12 | GTCATTGGTACATTCTTGCTGG | CACCATCCACCAAGACAAACT |
| Exon 13 | GGAAGTTCTAACAGAAATGATTTGG | GATGTAGGAGGTAAATGAATGTGCT |
| Exon 14 | GACCTAGGCCCTAAACTTTGG | GGTTCTTGAAATAGGAGGACCA |
| Exon 15 | AGAAGTTTGGAAGCATGTGCTG | CTCCCACAAATAACAAGTAGGTGC |
| Exon 16 | CATACAGATGCCACTCAAGAACC | GGCAGATTCTTTGTCTCTCTTCAT |
| Exon 17 | CTTGAGTGAGTAGTACATTTGCTCC | GTTAGGGAACACACCAGGTAATG |
| ***MLC1*** |  |  |
| Exon 1-2 | TGTAGTTGTCAGGGTGCCAGTG | GAGTTGCTCTCTCTGTCCCACC |
| Exon 3 | CAGTGAAGCAGAAGTTGAAGGGTC | GTGACAGAAACCTGCACATCTCA |
| Exon 4-5 | GATTCAGATCCTTCTGGAAGCG | CGAGCTGTGAATAACATTTACCAC |
| Exon 6-7 | CATGTGAGGCAGGAGAGTGTG | ACGTTTAATCCAGCCTCAGTCAC |
| Exon 8 | GCTCCTCCACGGTTAGATCTG | CTGTGGTGACTCTCTGTCTGAATG |
| Exon 9 | GTGGGAGGGCACATGTTTG | CCAGTCACTGCGCTCTCTTG |
| Exon 10 | AACCAGCTTGGGACTATGCC | CCAGAGTCCCAGGCAAAGC |
| Exon 11 | ATAGCAATGCGTTTGGGTTACTG | CCACTCACCTCCCCAGCTTC |
| Exon 12 | CCCACTTCCTTCACATTCGAA | GCAGTAGCTCAGGGCGATTAG |
| ***BTD*** |  |  |
| Exon 1 | GAATGTAAACACGCGCGTTC | CCACAAAGCGTGTAAGTAAATGC |
| Exon 2 | CGAGTGAGTTTAATTGCTGGGA | GTGGTAGGAAGCCTGGGATTAC |
| Exon 3 | CCTGCTACAGAGTAACTTCCTGATG | CTGCCTTGTAACGTCAGACATTC |
| Exon 4 | CTCACTGCAAGCTCCGCCTA | GGTTTAAGAGGAAGACACTGAGGG |
| ***GFAP*** |  |  |
| Exon 1 | ATCGCCAGTCTAGCCCACTC | CCTGAGACTTCTCGGGCACT |
| Exon 2-3-4 | CTCTGTGACAGCTGCCTCAAGG | GATATTCTCCCAGCTTCCTCCAC |
| Exon 5-6 | CTCACCTCCCTGATTTCCCAGA | TCAAACATCTAGTGACTGCCTGC |
| Exon 7 | GCGTTCAAGTTGTCATGATGGC | TCCCAGTCTGGAGCAACCTACA |
| Exon 8-9 | AGCCCGTTTGGAAGAGCCTAG | TTTCTTGTTAGTTGGAGTTGCTGG |
| ***GJC2*** |  |  |
| Exon 1 | TGTAGACAGATGGGTGGGAGAG | GTGCACCCTCCTATGGCTG |
| Exon 2 | CCACGTCATTGACTGTGTAAGC | TAACCAGCAGGAAGACCGTC |
|  | CAGTACCTGCTGTACGGCTTC | ATTTGCTGTTGCAGGGAATG |
| ***HEXB*** |  |  |
| Exon 1 | GAAATTCTCGAGGTGACCTGG | GTTTCTCGTCTGCGCAGTG |
| Exon 2 | GTTGGACTTACAATGGGCAGC | AACTCAGTGGTTCCTAGCATGG |
| Exon 3 | AGATGTACAGGAGGCAGTTAGAG | GCATTCTGTAAAGGATCTAAGGC |
| Exon 4-5 | TGCCTTACCTGGTTATGAGTCTG | GACCTCCACGTCACAGCTGA |
| Exon 6 | GGAAGCAGACATATTGGAAGC | CACCGTAACAATTACAGTAACCG |
| Exon 7 | ACAATTTCCAGGATCAAATCTACG | GGGTGACAGAACAAGACTCCA |
| Exon 8-9 | GATATAATGGGAAACAAAGAGGC | TACACTTAGGCAAAGGATGTAGAG |
| Exon 10-11 | CTCTCAAAATGCAAGAAATCC | AAGGTAACAATGGTTGCTTCAC |
| Exon 12-14 | TGCCTCTGTGTATAAGCTTTGA | GCCAGGCCTCTAAATGTAATAAG |
| ***HEXA*** |  |  |
| Exon 1 | ACATTTGTGTGATCTTGTGATCCTC | GGAAGTGGAGTGCCTGTGATCAG |
| Exon 2 | GCGTGTGAGCTGAGGGACTA | CCGAGCATCAGCAGTTTAGG |
| Exon 3 | AGGCCATCCTACAGCTGTCTTG | GGGTAAAACCTGTCAACTTCCAG |
| Exon 4-5 | GCTCTGCTACATTGAGAACCTTCC | CACCACACCCAGTCCATACATTC |
| Exon 6-7 | TAATTGAGAGCTGAGGCAGGTC | CTAAGGACCAAGGCTGGGATATG |
| Exon 8-9-10 | TGACTACCAGTGTCTGTGAGTGCC | CCAGGAGGATCAGTCTCTGTAGAGG |
| Exon 89 | CCACATGTAGGGCTTCTGAGATC | - |
| Exon 11-12-13 | GAGCACATACCTGTTGTCCCATG | CGTGGATGAGGGCTGACTATAAG |
| Exon | TGAGATTCCCAGGTGCATTTG | GGCTCCACTACCATTCACCTACAG |
| ***ASPA*** |  |  |
| Exon 2 | CTGACCATCTGAGGGAGTTAG | TACGACTGCATGTACGGAC |
| Exon 3 | GTCCTATTCATGACCAGCCAC | ACTGCCTCTGTGTTCTGGTAGC |
| Exon 4 | AGGTATTATTGACTCTGTTGAAGC | CATGGTGAAACTCCGTCTGTAC |
| Exon 5 | CCAGTCTTTATTCACTATGCATC | TTTCTTGAATGTGTCTATCCTGG |
| Exon 6 | TTGTTGCCCAGGTTGGAGT | TGTACGCGAAGTGCTGTATG |
| Exon 7 | TCAATGCCTCGCTCAAGTATC | AGACTTTCTAGACACAGGGTTGC |
| ***SLC17A5*** |  |  |
| Exon 1 | CAATCCTACGAGAACTCCCAGAACT | CTGCACAATGTCAAGATATCAGCTG |
| Exon 2 | CATGCCGTATATCTTTGCCAGC | AAGTTATTGCCCAATCAGTTCCTG |
| Exon 3 | GAAAGTTTGCATGTTCTTCAGTCC | GGGTGCTCCTACTAGAGACCTCA |
| Exon 4 | ACCTGATTCGTTTGATTCTTATCC | ATCCAACATTGCATCGTTCTG |
| Exon 5 | CCCATCCTCTGTAAGCAGTAGAC | TGTGGTTCAGTGATTTGGAAGAT |
| Exon 6 | AATTGAATCCAAACTGAAGTCAG | TGTCTAGAGCATGCACAATAGG |
| Exon 7 | GCTGATACGGTACAGATCTAAGC | TGTCTGGATACTAGACCCTTATTAG |
| Exon 8 | CCTATAATCCTTCTGACAGCCAG | CAGAACTGGTTATGTGTAGGCAG |
| Exon 9 | TGTTTCTACAGTAGGTGGCAGCA | CGCCTCTGGCCTTTTATCAG |
| Exon 10 | GGAAAGAATGAAGCTGTTTAAATG | CCATCCATTAAGGCATTTAGCT |
| Exon 11 | GATGTTGTAGCCCATTCTCATTG | CGACTAGCAGGCAGGTATGTG |
| ***FAM126A*** |  |  |
| Exon 2 | AGGCTTGTTTGTGCACATTG | CTTCCACAGATATTTAGCCTTTCAG |
| Exon 3 | CTAATCTTCTTCTCCCATCTGCAG | CAGAGATGGCTTCTGGAATACG |
| Exon 4-5-6-7 | GTAACTACCCAGGATTCCCATTC | GGGTAGGCTGAGCTTTACATTAAC |
| Exon 8 | TACCTAGTACATTGTTGGCTTATA | GTATGCAAATCACTTATCTATTGAC |
| Exon 9-10 | GGCATTTAGATGCAATTGAAAGG | GCAACTCCACAGCTCTTTCTTC |
| Exon 11 | CTTAATGGGGTATGGTTTATGGC | GGAATACGGATGAAAGACCCAG |
| ***ABCD1*** |  |  |
| Exon 1 | TGGAGGAGTCACTGTCGCTTC | AGCCTTCCTGCCTCTCCACT |
|  | CCACATACACCGACAGGAAGG | TATGGAGCCCACAAAGTCTACCC |
| Exon 2 | ATCGTAACCTCTGGCTCTCG | GTAACAAAGGGCGTGAGGTG |
| Exon 3-4 | TGACTTTCCGCTGTCTCTGC | GAGCGGGAATAGGAGGAGCT |
| Exon 5 | CTGAAGATCCGAGGTAAGGCTG | ACGTACATCTAGGGACAATGGCT |
| Exon 6-7 | CTCTCAAGGCTGGTCAGGAG | CTGGCACTTTAGACTCTGGATG |
| Exon 8-9-10 | CATGATTAATGCCTGTCAGACAGAC | GAATCATGGGGACTCGAGTCTC |
|  | TAGGGCTTGGACTCCACCG | TCCACATCTACTTTCCCGAGGG |
| ***PLP1*** |  |  |
| Exon 1-2 | GTTTTTGACTCTGAGCCCCTGT | CACGACTCATTTAAACCATGGAAC |
| Exon 3-4 | ACTATCTCCGAGCCTGTGAGCAC | CATCGCTTCCTCACTCAAATGC |
| Exon 5-6 | CCCAGTTTGTGTTTCTACATCTGC | CACACTTAGCCAACATGTTGTAGAAG |
| Exon 7 | TACACTGAAGACTGGGAGGC | TCAAGGATGGAAGCAGTCTACC |
| Exon 8 | GTCAAGTGTATATGGAGAAAGCC | GAGAAGATGCTGACAACACCC |

|  |  |
| --- | --- |

Table 2: The 59 genes included in panel gene analysis

| **Gene** | **NM no.** | **MIM** | **Gene** | **NM no.** | **MIM** | **Gene** | **NM no.** | **MIM** |
| --- | --- | --- | --- | --- | --- | --- | --- | --- |
| *ABCD1* | NM_000033.3 | 300371 | *GALC* | NM_000153 | 606890 | *PEX26* | NM_001127649 | 608666 |
| *ACOX1* | NM_001185039 | 609751 | *GBE1* | NM_000158 | 607839 | *PEX3* | NM_003630 | 603164 |
| *ALDH3A2* | NM_000382 | 609523 | *GFAP* | NM_002055 | 137780 | *PEX5* | NM_001131024 | 600414 |
| *ARSA* | NM_000487.5 | 607574 | *GJA1* | NM_000165 | 121014 | *PEX7* | NM_000288 | 601757 |
| *ASPA* | NM_001128085.1 | 608034 | *GJC2* | NM_020435 | 608803 | *PEX6* | [NM_000287](http://www.ncbi.nlm.nih.gov/nuccore/NM_000287) | 601498 |
| *CSF1R* | NM_005211 | 164770 | *HEPACAM* | NM_152722 | 611642 | *POLR3A* | NM_007055 | 614258 |
| *CYP27A1* | NM_000784 | 606530 | *HSD17B4* | NM_001199291 | 601860 | *PLP1* | NM_001128834 | 300401 |
| *DARS2* | NM_018122.4 | 606530 | *HTRA1* | NM_002775 | 602194 | *PSAP* | NM_002778 | 176801 |
| *EARS2* | NM_001083614 | 612799 | *PEX2* | [NM_001172087](http://www.ncbi.nlm.nih.gov/nuccore/NM_001172087) | 170993 | *POLR3B* | NM_018082 | 614366 |
| *EIF2B1* | [NM_001414](http://www.ncbi.nlm.nih.gov/nuccore/NM_001414) | 606686 | *SAMHD1* | NM_015474 | 606754 | *SAMHD1* | NM_015474 | 606754 |
| *EIF2B2* | [NM_014239](http://www.ncbi.nlm.nih.gov/nuccore/NM_014239) | 606454 | *RNASEH2A* | NM_006397 | 606034 | *SLC17A5* | NM_012434 | 604322 |
| *EIF2B3* | NM_020365 | 606273 | *RNASEH2C* | NM_032193 | 610330 | *SOX10* | NM_006941 | 602229 |
| *EIF2B4* | NM_001034116 | 606687 | *RNASEH2B* | NM_024570 | 610326 | *SUMF1* | NM_182760 | 607939 |
| *EIF2B5* | NM_003907 | 603945 | *TREX1* | NM_033629 | 606609 | *TREX1* | NM_033629 | 606609 |
| *FAM126A* | [NM_032581](http://www.ncbi.nlm.nih.gov/nuccore/NM_032581) | 610531 | *TYROBP* | NM_003332 | 604142 | *L2HGDH* | NM_024884 | 609584 |
| *FUCA1* | [NM_000147](http://www.ncbi.nlm.nih.gov/nuccore/NM_000147) | 612280 | *TREM2* | NM_018965 | 605086 | *MLC1* | NM_015166 | 605908 |
| *NOTCH3* | NM_000435 | 600276 | *PEX10* | NM_153818 | 602859 | *PEX13* | NM_002618 | 601789 |
| *PEX1* | NM_000466 | 602136 | *PEX12* | NM_000286 | 601758 | *PEX14* | NM_004565 | 601791 |
| *PEX16* | NM_057174 | 603360 | *PEX19* | NM_002857 | 600279 |  |  |  |

Table 3- The 114 patients genetically defined diagnosis with clinical characteristics, genes and variants

| No. | Gene | Phenotype | Age of onset (years) ♠ | sex | ethnicity | consanguinity | Age of onset | Nucleotide change | AA change | zygosity | Segregation | Medical examination | Clinical manifestation | Developmental milestone | Physical examination/Brain MRI | Ref |
| --- | --- | --- | --- | --- | --- | --- | --- | --- | --- | --- | --- | --- | --- | --- | --- | --- |
|  | *FAM126A* | HCC | 5 | M | Turk | FC | EJ | c.415-1G>A | - | Hom | Ma:Het  Pa:Het | - |  | / | congenital cataract/hypomyelination | [[1](#_ENREF_1)] |
|  | *POLR3A* | 4H | 3 | F | Fars | FC | EJ | c.2423G>A | p.Arg808Gln | Hom | NA |  | Developmental delay | hypotonia, unable to walk, seizure at 4 month, speech problem nystagmus, ataxia | hypomyelination | [[8](#_ENREF_2)] |
|  | *POLR3B* | 4H | 13 | M | Turk | FC | LJ | c.2099A>C | p.Asn700Thr | Hom | Ma:Het  Pa:Het | - | Motor regression | walking problems, tremor, movement problems | Tremor, ataxia, hypotonia/white matter abnormal; cerebrall atrophy, hypoplasia of the corpus callosum (HCAHC). | This study |
|  | *SLC17A5* | Salla | 6 | M | Lur | FC | EJ | c.786A>C | p.Gln262Asp | Hom | Ma:Het  Pa:Het | ↑ free sialic acid | Developmental delay, motor delay | seizure, no speech | seizure, spasticity/ hypomyelination | This study |
|  | *GFAP* | AxD | 1.6 | M | Kurd | NC | I | c.715C>T | p.Arg239Cys | Het | Ma:N  Pa:N |  | Motor retardation | hypotonia, developmental delay, seizure | hypotonia/demyelization of brain | [[1](#_ENREF_3)] |
|  | *ABCD1* | X-ALD | 10 | M | Arab | NC | J | c.904_905delinsAT | p.Glu302Met | Hemi | NA | VLCFA assay: C26:0 ↑ | Cognition impairment | seizure, speech problems |  | This study |
|  | *ABCD1* | X-ALD | 7 | M | Afghan | C | J | c. 1415_1416delAG | p.Gln472Arg fs*83 | Hemi | NA | VLCF↑ | cognition impairment | learning difficulty, attention deficient, |  | [[2](#_ENREF_4)] |
|  | *ABCD1* | X-ALD | 3 | M | Fars | NC | J | c. 1415_1416delAG | p.Gln472Arg fs*83 | Hemi | NA | VLCF↑ | Cognition impairment | walking difficulty, vomiting, cognition impairment | impaired adrenocortical function | [[2](#_ENREF_4)] |
|  | *ABCD1* | X-ALD | 7 | M | Fars | NC | J | c.1628C>G | p.Pro543Arg | Hemi | Ma:Het  Pa:N | VLCF↑ | Cognition impairment | cognition impairment | FH of Addison | This study |
|  | *ABCD1* | X-ALD Addison | 7 | M | Fars | C | J | c.1814T>C | P.Leu605Pro | Hemi | Ma:Het | VLCF↑ |  | increased pigment in knees, elbow, Gums and teeth (hyperpigmentation), Diarrhea and vomiting, seizures, irritability | impaired adrenocortical function |  |
|  | *ABCD1* | X-ALD | 5 | M | Turk | NC | J | c.2002A>G and c.1021G>T | p.Thr668Ala and p.Ala341Ser | Hemi | Ma:Het | C24/C22 1.93 and C26/C22 0.092 | Motor regression | leg stiffness, weakness in muscle, spasms and weakness | impaired adrenocortical function | This study |
|  | *ABCD1* | X-ALD | 7 | M | Turk | FC | J | c.839G>C | p.Arg280Pro | Hemi | Ma:Het | VLCF↑ |  | vomiting | impaired adrenocortical function | This study |
|  | *ABCD1* | X-ALD | 20 | M | Mazani | NC | A | c.1850G>A | p.Arg617His | Hemi | NA | C24/C22 1.96 and C26/C22 0.146 both of them are high | Motor regression | walking difficulty and weakness of legs, vision problem | impaired adrenocortical function | [[2](#_ENREF_6)] |
|  | *RNASET2* | RNAse T2 deficiency | 7m | F | Fars | FC | I | c.233C>A | p.Ser78Ter | Hom | Ma:Het  Pa:Het |  | Developmental delay | Hypotonia | Hypotonia, CT calcification, brain atrophy, | This study |
|  | *ASPA* | CNV | 2m | M | Fars | C | I | c.914C>A and c.237_238insA | p.Ala305Glu and p.Met82AsnfsTer8 | compound het | Ma:Het c.237_238insA  Pa:Het c.914C>A | ↑ N-acetylaspartic acid in urine | Motor retardation, Developmental delay | Improved nystagmus | Nystagmus, Macrocephaly | [[1](#_ENREF_7),[4](#_ENREF_8)] |
|  | *ASPA* | CNV | 1 | M | Lur | FC | I | c.634+1G>T | - | Hom | Ma:Het  Pa:Het | ↑ N-acetylaspartic acid in urine | Developmental delay | Hypotonia, irritable | Macrocephaly, hypotonia | [[1](#_ENREF_9)] |
|  | *ASPA* | CNV | 6m | M | Turk | NC | I | c.237_238insA | p.Met82Asnfs*8 | Hom | NA | ↑ N-acetylaspartic acid in urine | Motor retardation | hypotonia, irritable | Macrocephaly, hypotonia | [[4](#_ENREF_8)] |
|  | *ASPA* | CNV | 2m | M | Kurd | C | I | c.437_449delCTCTGGCTCCACT | p.Ser146TyrfsX7 | Hom | Ma:Het  Pa:Het |  | Developmental delay | irritable, eye movements problem | Macrocephaly, hypotonia, | This study |
|  | *ASPA* | CNV | 3m | M | Fars | FC | I | c.634+1G>T | - | Hom | Ma:Het  Pa:Het |  | motor retardation | Developmental delay, speech problem | Macrocephaly, spasticity, high Deep Tendon Reflexes, hypotonia | [[1](#_ENREF_9)] |
|  | *ASPA* | CNV | 8m | M | Fars | FC | I | c.914C>A | p.Ala305Glu | Hom | NA |  | motor retardation, mental retardation | muscle weakness, deviation of the eyes | hypotonia | [[1](#_ENREF_7)] |
|  | *ASPA* | CNV | 3m | M | Arab | FC | I | c.634+1G>T | - | Hom | Ma:Het  Pa:Het |  | Developmental delay | muscle weakness, feeding and swallowing difficulties, hypotonia, irritable | Macrocephaly, Developmental delay | [[1](#_ENREF_9)] |
|  | *ASPA* | CNV | 4m | F | Afghan | FC | I | c.634+1G>T | - | Hom | NA |  | Developmental delay | muscle weakness, hypotonia, breathing difficulties, seizure | Developmental delay Macrocephaly, | [[1](#_ENREF_9)] |
|  | *ASPA* | CNV | 1d | F | Mazani | FC | N | c.359C>T | p.Ser120phe | Hom | NA |  |  | hypotonia, irritable |  | This study |
|  | *ASPA* | CNV | 1d | M | Arab | FC | N | c.634+1G>T | - | Hom | Ma:Het  Pa:Het | ↑ N-acetylaspartic acid in urine; |  | hypotonia, irritability, vision problems, behavior problems, loss of the ability to fix and follow with the eyes | Nystagmus, Macrocephaly | [[1](#_ENREF_9)] |
|  | *ASPA* | CNV | 1d | F | Lur | FC | N | c.634+1G>T | - | Hom | Ma:Het  Pa:Het |  | Developmental delay | Spasticity and seizure | Macrocephaly, Developmental delay | [[1](#_ENREF_9)] |
|  | *ASPA* | CNV | 3m | F | Turk | FC | I | c.237_238insA | p.Met82Asnfs*8 | Hom | NA | ↑ N-acetylaspartic acid in urine; | Developmental delay | Seizure, hypotonia, learning difficulties, spasticity , vision problems, irritable, loss of ability to fix and follow with the eyes | hypotonia, high Deep Tendon Reflexes (DTR, nystagmus | [[4](#_ENREF_8)] |
|  | *EIF2B3* | VWM | 2 | F | Turk | FC | LI | c.674G>A | p.Arg225Gln | Hom | NA |  | Motor regression |  |  | [[2](#_ENREF_10)] |
|  | *EIF2B5* | VWM | 9m | M | Fars | FC | I | c.1015C>T | p.Arg339Trp | Hom | NA |  | Motor regression | Seizure, tremor, bristling head, sitting problem, loss of the ability to fix and follow with the eyes | Hypotonia, high Deep Tendon Reflexes (DTR) | [[4](#_ENREF_11)] |
|  | *EIF2B5* | VWM | 2 | F | Fars | FC | LI | c.407G>A | p.Arg136His | Hom | Ma:Het  Pa:Het |  | Motor regression | seizure, speech problem, walking difficulty, uncontrollable movement |  | [[1](#_ENREF_12)] |
|  | *EIF2B4* | VWM | 2 | F | Fars | FC | LI | c.866C>T | p.ser289Ile | Hom | NA |  | Motor regression | Walking problem | NA | This study |
|  | *PPT1* | NCL | 4 | M | Balooch | FC | EJ | c.362+5G>A | inronic | Hom | Ma:het  Pa: het |  | Developmental delaly, motor regression | No walking and no speech from 3 years of age | Hypontonia, brain atrophy, optic atrophy, myoclonus |  |
|  | *CLN6* | NCL | 4 | F | Balooch | FC | EJ | c.659A>C | p.Tyr220Ser | hom | Ma:het  Pa:Het |  | Motor regression | Seizure, walking difficulty, speech problem | Cortex atrophy, myoclonic seizure, tremor |  |
|  | *FUCA1* | fucosidosis | 2 | F | Arab | TC | LI | c.422G>T | p.Gly141Val | Hom | NA |  | Development delay | skin lesions, walking problem, atrophy, | Dental germination disorder, Gap junction protein (ODDD), Calcification, hypotonia, hypomyelination | This study |
|  | *FUCA1* | fucosidosis | 3 | F | Fars | FC | EJ | c.82delG | p.Val28CysfsX105 | Hom | Ma:Het/  Pa:Het |  | Developmental delay | developmental delay, speech problem | delayed myelination, atrophy | This study |
|  | *GALC* | Krabbe | 3m | F | Lur | C | I | c.830G>A | p.Ser277Asn | Hom | Ma:Het  Pa:Het |  | Motor regression | dystonia, spasticity hypotonia |  | This study |
|  | *GALC* | Krabbe | 7m | M | Fars | SC | I | c.1942A>T | p.Lys648Ter | Hom | Ma:Het  Pa:Het |  | Motor regression | difficulty of speech, swallowing | ataxia, developmental delay | This study |
|  | *GALC* | Krabbe | 2 | M | Fars | FC | EJ | c.787G>A | p.Ala263Thr | Hom | Ma:Het  Pa:Het |  | Motor regression | seizure, speech problem, walking difficulty |  | [[4](#_ENREF_13)] |
|  | *GALC* | Krabbe | 6 | F | Kurd | SC | I | c.1901_1901delT | p.Leu634Ter | Hom | Ma:Het  Pa:Het | Deficient galactocerebrosidase activity | Motor regression, developmental delay | Spasticity |  | [[4](#_ENREF_13)] |
|  | *GALC* | Krabbe | 3 | M | Fars | NC | EJ | EX11_17del and of c.334A>G | del+p.Thr112Ala | compund het | Ma:Het DEL  Pa:Het |  | Motor regression | Seizure, speech problem, walking difficulty | white vanishing matter | [[1](#_ENREF_14)] and This study |
|  | *L2HGDH* | L-2-HGA | 10 | M | Kurd | FC | LJ | c.408+1G>C | - | Hom | Ma:Het  Pa:Het | ↑ concentration of L-2hydroxyglutaric acid | Mental retardation | walking problems, speech problem, tremor |  | This study |
|  | *L2HGDH* | L-2-HGA | 12 | M | Kurd | NC | LJ | c.905C>T | p.Pro302Leu | Hom | NA | ↑ concentration of L-2hydroxyglutaric acid | Mental retardation | Seizure, learning problem | Macrocephaly, Subcortical denta | [[22](#_ENREF_15)] |
|  | L2HGDH | L-2-HGA | 5 | M | Fars | FC | EJ | c.1213A>G | p.Arg405Gly | Hom | NA | ↑ concentration of L-2hydroxyglutaric acid | Mental retardation | learning problem, hypotonia |  | This study |
|  | *MLC1* | MLC | 6m | M | Turk | FC | I | c.449_455delTCCTGCT | p.leu150Argfs*9 | Hom | Ma:Het  Pa:Het |  | Motor delay | impaired speech (dysarthria), dystonia | Macrocephaly, MRI: cystic lesions, | [[3](#_ENREF_16)] |
|  | *MLC1* | MLC | 3 | M | Turk | FC | EJ | c.449_455delTCCTGCT | p.leu150Argfs*9 | Hom | Ma:Het  Pa:Het |  | Motor delay | walking difficulty, dystonia | Macrocephaly, Megalencephalic leukodystrophy w/ subcortical cysts | [[3](#_ENREF_16)] |
|  | *MLC1* | MLC | 7 | M | Turk | NC | LJ | c.177+1G>T | - | Hom | Ma:Het  Pa:Het |  | Motor retardation | Walking difficulty, seizure | Megalencephalic leukodystrophy w/ subcortical cysts | [[4](#_ENREF_17)] |
|  | *MLC1* | MLC | 15 | F | Turk | C | LJ | c.177+1G>T | - | Hom | Ma:Het  Pa:Het |  | Motor delay | late walking, loss of walking, Muscle weakness | Megalencephalic leukodystrophy w/ subcortical cysts | [[4](#_ENREF_17)] |
|  | *MLC1* | MLC | 9 | F | Kurd | NC | LJ | c.183C>A | p.Cys61Ter | Hom | Ma:Het  Pa:Het |  | Motor retardation | walking difficulty, seizure, spasticity | Megalencephalic leukodystrophy w/ subcortical cysts | This study |
|  | *MLC1* | MLC | 9m | M | Turk | FC | I | c.819C>G | p.Phe273Leu | Hom | Ma:Het  Pa:Het |  | Motor delay |  | Megalencephalic leukodystrophy w/ subcortical cysts, 2 black spots, | This study |
|  | *MLC1* | ML2LC | 6m | F | Turk | NC | I | c.449_455delTCCTGCT and c.-42C>T | p.leu150Argfs*9 | compound Het | Ma: het c.-42C>T  Pa: het c.449_455delTCCTGCT |  | Motor delay |  | Megalencephalic leukodystrophy w/ subcortical cysts | [[3](#_ENREF_16),[2](#_ENREF_18)] |
|  | *SUMF1* | MSD | 3 | F | Turk | FC | LI | c.739G>C | p.Gly247Arg | Hom | NA |  | Motor and mental retardation | dried skin, spasticity, incapable of walking and talking | coarse facial feature, ichtyosis; Atrophy, demyelination | [[4](#_ENREF_19)] |
|  | *PLP1* | PMD | 4 | M | Fars | NC | EJ | c.EX1_7 DEL | Del | Hemi | NA |  | Motor regression | nystagmus, hypotonia | white matter abnormal; | [[6](#_ENREF_20)] |
|  | *GJC2* | PMLD | 2 | M | Arab | TC | I | c.571_572insC | p.Thr195AspfsX69 | Hom | Ma:Het  Pa:Het |  |  | ataxia, nystagmus, and hypotonia | hypomyelination | This study |
|  | *GJC2* | PMLD | 5 | M | Fars | NC | EJ | c.118G>C | p.Ala40Pro | Hom | NA |  |  | nystagmus | hypomyelination | This study |
|  | *GJC2* | PMLD | 5 | F | Arab | TC | EJ | c.118G>C | p.Ala40Pro | Hom | NA |  | Development delay | nystagmus, problems producing speech, develop skeletal issues, and weak muscle tone | hypomyelination | This study |
|  | *GJC2* | PMLD | 6 | F | Fars | TC | EJ | c.733T>A | p.Cys245Ser | Hom | Ma:Het  Pa:Het |  | Developmental delay | nystagmus, problems producing speech, develop skeletal issues, hypotonia | hypomyelination | This study |
|  | *GJC2* | PMLD | 6 | M | Lur | C | EJ | c.883C>T | p.Gln295Ter | Hom | NA |  | Developmental delay | ataxia, nystagmus, speech problem | hypomyelination | This study |
|  | *GJC2* | PMLD | 6 | M | Arab | TC | EJ | c.733T>A | p.Cys245Ser | Hom | NA |  | Developmental delay | ataxia, nystagmus and speech problem | hypomyelination | This study |
|  | *GJC2* | PMLD | 5 | M | Turk | TC | EJ | c.903_919del17 | p.Gly303Profs*39 | Hom | NA |  | Developmental delay | ataxia, nystagmus, speech problem, seizure | hypomyelination | [[2](#_ENREF_21)] |
|  | *GJC2* | PMLD | 6 | F | Fars | TC | EJ | c.970_971insGC | p.Ala325Profs | Hom | Ma:Het  Pa:Het |  | Developmental delay | nystagmus, speech problem, hypotonia | WM abnormality | [[10](#_ENREF_22)] |
|  | *PEX7* | RCDP | 1,8m | F | Kurd | NC | LI | c.370_396del27 | p.Gly124-Ser132del | Hom | Ma:Het  Pa:Het |  | Developmental delay, motor regression | cataract, digestive problem | coarse facial feature, syndromic | [[3](#_ENREF_23)] |
|  | *PEX13* | Zellweger | 2m | M | Turk | FC | N | c.529_531 del AAA | p.Lys177del | Hom | NA |  | Motor regression | seizure 2m, 6 m digestive problems, walk 2yrs, hypotonia infancy, hypodontia later 6yrs, vomiting | abnormal WM, demyelination | This study |
|  | *NAGLU* | MPS IIIB | 8 | M | Lur | FC | EJ | c.480delT | p.Asn160Lysfs | Hom | NA |  |  |  | coarse facial feature, MPSIII, macrocephaly |  |
|  | *HSD17B4* | DBPD | 37 | M | Arab | C | A | c.392C>A | p. Thr131Lys | Hom | NA |  | motor regression | swallowing problem, walking difficulty, speech problem, | hypomyelination |  |
|  | *RARS* | HLD9 | 10 | F | Fars | FC | LJ | c.2T>C | p.Met1Thr | Hom | NA |  | motor delay | spasity, hypotonia | hypomyelination | [[2](#_ENREF_25)] |
|  | *HEXB* | SHS | 2m | M | Mazani | FC | I | c. 850C>T | p.Arg284Ter | Hom | Ma:Het  Pa:Het | Assay | Developmental delay | sitting problem, vision loss, muscle weakness, breathing problem, exaggerated startle reaction to loud noises |  | [[1](#_ENREF_26)] |
|  | *HEXB* | SHS | 1 | M | Lur | FC | I | c.833C>T | p.Ala278Val | Hom | Ma:Het  Pa:Het | Assay | Developmental delay | metabolic problem, muscles weakness, motor skill problem |  | [[1](#_ENREF_27)] |
|  | *HEXB* | SHS | 4m | M | Arab | FC | I | c.833C>T | p.Ala278Val | Hom | Ma:Het  Pa:Het | Assay |  | Hearing problem, cherry red spot | cherry red spot | [[1](#_ENREF_27)] |
|  | *HEXB* | SHS | 7m | M | Turk | FC | I | c.1615C>T | p.Arg539Cys | Hom | NA | Assay | Developmental delay | muscles weakness, seizure |  | [[1](#_ENREF_28)] |
|  | *HEXB* | SHS | 3m | M | Fars | C | I | c.655_657delATT | p.Ile219del | Hom | Ma:Het  Pa:Het | Assay | Developmental delay | Muscle weakness, sitting problem |  | This study |
|  | *HEXB* | SHS | 1 | F | Fars | FC | I | c.1602C>A | p.Cys534Ter | Hom | NA | Assay |  |  |  |  |
|  | *HEXA* | Tay-Sachs | 6m | F | Gilak | FC | I | c.509G>A | p.Arg170Gln | Hom | Ma:Het  Pa:Het | Assay | Motor retardation | Sitting disability, nystagmus |  | [[5](#_ENREF_30)] |
|  | *HEXA* | Tay-Sachs | 6m | F | Turk | NC | I | c.986+3A>G and c.1495C>T | p.Arg499Cys | compound heterozygous | Ma: het c.986+3A>G, Pa: het c.1495C>T | Assay | Motor retardation | sitting and walking disability, muscle weakness |  | [[4](#_ENREF_31),[1](#_ENREF_32)] |
|  | *HEXA* | Tay-Sachs | 10m | F | Fars | FC | I | c.1385A>T | p.Glu462Val | Hom | Ma:Het  Pa:Het | Assay | Motor retardation | sitting disability, muscle weakness |  | [[6](#_ENREF_33)] |
|  | *HEXA* | Tay-Sachs | 6m | M | Fars | C | I | c.409C>T | p.Arg137Ter | Hom | - | Assay | Motor retardation | seizure, sitting disability, speech problem, muscle weakness |  | [[1](#_ENREF_34)] |
|  | *HEXA* | Tay-Sachs | 6m | M | Gilak | NC | I | c.509G>A | p.Arg170Gln | Hom | Ma:Het  Pa:Het | Assay | Motor retardation | Muscle weakness, walking problem, intellectual disability, vision problem | positive CRS | [[5](#_ENREF_30)] |
|  | *HEXA* | Tay-Sachs | 6m | F | Gilak | FC | I | c.754C>T | p.Arg252Cys | Hom | Ma:Het  Pa:Het | Assay | Developmental delay | Developmental delay, vision problem, seizure, disability to reaction to noises, |  | This study |
|  | *HEXA* | Tay-Sachs | 2 | M | Fars | C | I | c.1147-1G>T | - | Hom | Ma:Het  Pa:Het | Assay |  | Muscle weakness, nystagmus, |  | This study |
|  | *HEXA* | Tay-Sachs | 6m | M | Mazani | NC | I | c.1528C>T | p.Arg510Ter | Hom | Ma:Het  Pa:Het | Assay | Motor regression | muscle weakness, cherry red spot, sitting problem | cherry red spot, cerebral hypotonia | [[6](#_ENREF_35)] |
|  | *HEXA* | Tay-Sachs | 6m | M | Fars | C | I | c.1528C>T | p.Arg510Ter | Hom | - | Assay | Developmental delay | strabismus, developmental delay |  | [[6](#_ENREF_35)] |
|  | *HEXA* | Tay-Sachs | 17m | F | Turk | FC | I | c.1528C>T | p.Arg510Ter | Hom | Ma:Het  Pa:Het | Assay | Developmental delay | Sitting problem, speech loss, mental problem and ataxia | cherry red spot | [[6](#_ENREF_35)] |
|  | *HEXA* | Tay-Sachs | 7m | M | Turk | C | I | c.1528C>T | p.Arg510Ter | Hom | - | Assay | Developmental delay | Speech problem, seizure, nystagmus, muscles weakness | nystagmus, hypotonia | [[6](#_ENREF_35)] |
|  | *PLA2G6* | INAD | 4m | M | Fars | FC | I | c.16C>T | p.Arg6Cys | Hom |  | normal Phe levels |  | Seizure, hypotonia, bristling head, | brain progressive atrophy | This study |
|  | *GLB1* | Gangliosidosis | 1 | F | Turk | FC | LI | c.416T>A | p.Leu139Gln | Hom |  | Assay |  |  | Gangliosidosis | This study |
|  | *BTD* | BTD | 2m | F | Turk | SC | I | c.235C>T | p.Arg79Cys | Hom |  |  |  | Seizure |  | [[2](#_ENREF_36)] |
|  | *NDUFS7* | Mitochondrial complex I disorders | 2 | F | Fars | FC | LI | c.415G>A | p.Asp139Asn | Hom | Ma:het  Pa:het |  |  | Hypotonia, seizure | Hypotonia |  |
|  | *NDUFA5,*  *NDUFS1* | Mitochondrial complex I disorders | 2 | M | Mazani | FC | LI | c.826C>G,  c.1285G>A | p.Arg276Gly,  p.Val429Met | homo | Ma:het  Pa:het |  | Motor regression | Seizure, Walking problem |  |  |
|  | *SUCLA2* | Mitochondrial DNA depletion syndrome 5 | 4m | M | Fars | FC | I | c.997G>T | p.Asp333Tyr | Homo | Ma:het  Pa: het |  | Developmental delay | Muscle weakness | Developmental delay, dystonia | This study |
|  | *ERCC6* | CS | 5 | M | Kurd | FC | EJ | c.2203C>T, c.2551T>A | p.Arg735Ter, p.Trp851Arg | compound heterozygous | Pa:p.Trp851Arg,  Ma: p.Arg735Ter, |  | Motor regression, developmental delay |  | Microcephaly, hypomyelination | [[1](#_ENREF_37)] |
|  | *POLG* | ataxia neuropathy spectrum | 5 | F | Arab | FC | EJ | c.3482+6C>T | - | Hom | Ma:het  Pa: het |  | Developmental delay | Speech difficulty, walking difficulty, vision problem, | Cerebral ataxia | This study |
|  | *POLG* | ataxia neuropathy spectrum | 4 | M | Lak | FC | EJ | c.3286C>T | p.Arg1096Cys | Hom | Ma:het  Pa: het |  |  | Seizure |  | [[8](#_ENREF_38)] |
|  | *ETFDH* | Glutaric acidemia IIC | 8 | F | Fars | FC | LJ | c.1130T>C | p. Leu377Pro | Hom | Ma:het  Pa: het  Bro: N |  | Motor regression | Walking problem, speech problem, digestive problem |  | [[Pt 8](#_ENREF_39)] |
|  | *SDHAF1* | MCIID | 2 | M | Fars | FC | I | c.29A>C | p.Gln10Pro | Hom | Ma:het  Pa: het  Bro: N |  | Motor retardation | Speech problem, no walking |  | This study |
|  | *SURF1* | Leigh syndrome | 6m | F | Fars | NC | I | c.751C>T | p.Gln251Ter | Hom | Ma:het  Pa: het | Mitochondrial IV | Motor retardation | No walking, digestive problem |  | [[6](#_ENREF_40)] |
|  | *SURF1* | Leigh syndrome | 8m | F | Turkeman | FC | I | c.792_793delAG | p.Arg155Serfs | Hom | Ma:het  Pa: het  2uncles:het | Mitochondrial IV | Developmental delay, motor retardation | Muscle weakness, developmental delay, walking difficulty, sitting problem, no speech |  | [[6](#_ENREF_41)] |
|  | *SURF1* | Leigh syndrome  Mitochondrial complex IV disorders | 1 | F | Turk | FC | LI | c.808_812delGAGCA | p.Glu270SerfsX20 | Hom | Ma:het  Pa: het | Mitochondrial IV | Motor retardation | Muscle weakness, walking problem, swallowing problem |  | This study |

FC=first cousins, N=no; C= consanguineous, NA=not available

M=male, F=female, Pa=paternal, Ma=Maternal, Hom=homozygote, Het=heterozygote,

EJ=early Juvenile, LJ=late Juvenile, I= infantile, ♠ m=months, d=days

HCC= hypomyelination and congenital cataract, 4H=Hypodontia and/or hypogonadotropic hypogonadism, Salla disease=Free sialic acid storage disorders, AD= Alexander disease, XALD=X-linked adrenoleukodystrophy, CNV= Canavan disease, MLC= Megalencephalic leukoencephalopathy with subcortical cysts, VWM= Leukoencephalopathy with vanishing white matter (VWM disease), L-2-HGA= L-2-hydroxyglutaric aciduria disease, MSD=Multiple sulfatase deficiency, PMD= Pelizaeus-Merzbacher disease, PMLD= Pelizaeus-Merzbacher Like Disease, RCDP= Rhizomelic chondrodysplasia punctata, MPS IIIB= Mucopolysaccharidosis type IIIB, DBPD= D-bifunctional protein deficiency, MCIID=Mitochondrial complex II deficiency, CS= Cockayne syndrome, BTD= Biotinidase deficiency , RNAse T2 deficient leukoencephalopathy , NCL- Infantile neuronal ceroid lipofuscinosis, infantile neuroaxonal dystrophy/atypical neuroaxonal dystrophy =INAD

Hypomyelinating leukodystrophy-9= HLD-9

peroxisome biogenesis disease

1. Traverso M, Yuregir OO, Mimouni-Bloch A, Rossi A, Aslan H, Gazzerro E, et al. Hypomyelination and congenital cataract: identification of novel mutations in two unrelated families. Eur J Paediatr Neurol 2013; 17: 108-111.

2. Finegold DN, Baty CJ, Knickelbein KZ, Perschke S, Noon SE, Campbell D, et al. Connexin 47 mutations increase risk for secondary lymphedema following breast cancer treatment. Clin Cancer Res 2012; 18: 2382-2390.

3. Brenner M, Johnson AB, Boespflug-Tanguy O, Rodriguez D, Goldman JE, Messing A. Mutations in GFAP, encoding glial fibrillary acidic protein, are associated with Alexander disease. Nat Genet 2001; 27: 117-120.

4. Kemp S, Ligtenberg MJ, van Geel BM, Barth PG, Wolterman RA, Schoute F, et al. Identification of a two base pair deletion in five unrelated families with adrenoleukodystrophy: a possible hot spot for mutations. Biochem Biophys Res Commun 1994; 202: 647-653.

5. Cooper EVB, P.D. Stenson, A.D. Phillips, K. Evans, S. Heywood, M.J. Hayden, M.M. Chapman, M.E Mort, L. Azevedo and D.S. Millar. The Human Gene Mutation Database (HGMD®) represents an attempt to collate all known (published) gene lesions responsible for human inherited disease and is maintained in Cardiff by D.N.

6. Fanen P, Guidoux S, Sarde CO, Mandel JL, Goossens M, Aubourg P. Identification of mutations in the putative ATP-binding domain of the adrenoleukodystrophy gene. J Clin Invest 1994; 94: 516-520.

7. Kaul R, Gao GP, Aloya M, Balamurugan K, Petrosky A, Michals K, Matalon R. Canavan disease: mutations among Jewish and non-Jewish patients. Am J Hum Genet 1994; 55: 34-41.

8. Elpeleg ON, Shaag A. The spectrum of mutations of the aspartoacylase gene in Canavan disease in non-Jewish patients. J Inherit Metab Dis 1999; 22: 531-534.

9. Rady PL, Penzien JM, Vargas T, Tyring SK, Matalon R. Novel splice site mutation of aspartoacylase gene in a Turkish patient with Canavan disease. Eur J Paediatr Neurol 2000; 4: 27-30.

10. van der Knaap MS, Leegwater PA, Konst AA, Visser A, Naidu S, Oudejans CB, et al. Mutations in each of the five subunits of translation initiation factor eIF2B can cause leukoencephalopathy with vanishing white matter. Ann Neurol 2002; 51: 264-270.

11. Leegwater PA, Vermeulen G, Konst AA, Naidu S, Mulders J, Visser A, et al. Subunits of the translation initiation factor eIF2B are mutant in leukoencephalopathy with vanishing white matter. Nat Genet 2001; 29: 383-388.

12. Kantor L, Harding HP, Ron D, Schiffmann R, Kaneski CR, Kimball SR, Elroy-Stein O. Heightened stress response in primary fibroblasts expressing mutant eIF2B genes from CACH/VWM leukodystrophy patients. Hum Genet 2005; 118: 99-106.

13. Wenger DA, Rafi MA, Luzi P. Molecular genetics of Krabbe disease (globoid cell leukodystrophy): diagnostic and clinical implications. Hum Mutat 1997; 10: 268-279.

14. Luzi P, Rafi MA, Wenger DA. Multiple mutations in the GALC gene in a patient with adult-onset Krabbe disease. Ann Neurol 1996; 40: 116-119.

15. Topcu M, Jobard F, Halliez S, Coskun T, Yalcinkayal C, Gerceker FO, et al. L-2-Hydroxyglutaric aciduria: identification of a mutant gene C14orf160, localized on chromosome 14q22.1. Hum Mol Genet 2004; 13: 2803-2811.

16. Leegwater PA, Boor PK, Yuan BQ, van der Steen J, Visser A, Konst AA, et al. Identification of novel mutations in MLC1 responsible for megalencephalic leukoencephalopathy with subcortical cysts. Hum Genet 2002; 110: 279-283.

17. Patrono C, Di Giacinto G, Eymard-Pierre E, Santorelli FM, Rodriguez D, De Stefano N, et al. Genetic heterogeneity of megalencephalic leukoencephalopathy and subcortical cysts. Neurology 2003; 61: 534-537.

18. Kariminejad A, Rajaee A, Ashrafi MR, Alizadeh H, Tonekaboni SH, Malamiri RA, et al. Eight novel mutations in MLC1 from 18 Iranian patients with megalencephalic leukoencephalopathy with subcortical cysts. Eur J Med Genet 2015; 58: 71-74.

19. Dierks T, Dickmanns A, Preusser-Kunze A, Schmidt B, Mariappan M, von Figura K, et al. Molecular basis for multiple sulfatase deficiency and mechanism for formylglycine generation of the human formylglycine-generating enzyme. Cell 2005; 121: 541-552.

20. Matsufuji M, Osaka H, Gotoh L, Shimbo H, Takashima S, Inoue K. Partial PLP1 deletion causing X-linked dominant spastic paraplegia type 2. Pediatr Neurol 2013; 49: 477-481.

21. Karimzadeh P, Ahmadabadi F, Aryani O, Houshmand M, Khatami A. New mutation of pelizaeus--merzbacher-like disease; a report from iran. Iran J Radiol 2014; 11: e6913.

22. Henneke M, Combes P, Diekmann S, Bertini E, Brockmann K, Burlina AP, et al. GJA12 mutations are a rare cause of Pelizaeus-Merzbacher-like disease. Neurology 2008; 70: 748-754.

23. Motley AM, Brites P, Gerez L, Hogenhout E, Haasjes J, Benne R, et al. Mutational spectrum in the PEX7 gene and functional analysis of mutant alleles in 78 patients with rhizomelic chondrodysplasia punctata type 1. Am J Hum Genet 2002; 70: 612-624.

24. Yassaee VR, Hashemi-Gorji F, Miryounesi M, Rezayi A, Ravesh Z, Yassaee F, Salehpour S. Clinical, biochemical and molecular features of Iranian families with mucopolysaccharidosis: A case series. Clin Chim Acta 2017; 474: 88-95.

25. Rezaei Z, Hosseinpour S, Ashrafi MR, Mahdieh N, Alizadeh H, Mohammadpour M, et al. Hypomyelinating Leukodystrophy with Spinal Cord Involvement Caused by a Novel Variant in RARS: Report of Two Unrelated Patients. Neuropediatrics 2019; 50: 130-134.

26. Zhang ZX, Wakamatsu N, Mules EH, Thomas GH, Gravel RA. Impact of premature stop codons on mRNA levels in infantile Sandhoff disease. Hum Mol Genet 1994; 3: 139-145.

27. Tavasoli AR, Parvaneh N, Ashrafi MR, Rezaei Z, Zschocke J, Rostami P. Clinical presentation and outcome in infantile Sandhoff disease: a case series of 25 patients from Iranian neurometabolic bioregistry with five novel mutations. Orphanet J Rare Dis 2018; 13: 130.

28. Gort L, de Olano N, Macias-Vidal J, Coll MA, Spanish GMWG. GM2 gangliosidoses in Spain: analysis of the HEXA and HEXB genes in 34 Tay-Sachs and 14 Sandhoff patients. Gene 2012; 506: 25-30.

29. Mahdieh N, Mikaeeli S, Tavasoli AR, Rezaei Z, Maleki M, Rabbani B. Genotype, phenotype and in silico pathogenicity analysis of HEXB mutations: Panel based sequencing for differential diagnosis of gangliosidosis. Clin Neurol Neurosurg 2018; 167: 43-53.

30. Nakano T, Nanba E, Tanaka A, Ohno K, Suzuki Y, Suzuki K. A new point mutation within exon 5 of beta-hexosaminidase alpha gene in a Japanese infant with Tay-Sachs disease. Ann Neurol 1990; 27: 465-473.

31. Mules EH, Hayflick S, Miller CS, Reynolds LW, Thomas GH. Six novel deleterious and three neutral mutations in the gene encoding the alpha-subunit of hexosaminidase A in non-Jewish individuals. Am J Hum Genet 1992; 50: 834-841.

32. Richard MM, Erenberg G, Triggs-Raine BL. An A-to-G mutation at the +3 position of intron 8 of the HEXA gene is associated with exon 8 skipping and Tay-Sachs disease. Biochem Mol Med 1995; 55: 74-76.

33. Mistri M, Tamhankar PM, Sheth F, Sanghavi D, Kondurkar P, Patil S, et al. Identification of novel mutations in HEXA gene in children affected with Tay Sachs disease from India. PLoS One 2012; 7: e39122.

34. Akli S, Chelly J, Lacorte JM, Poenaru L, Kahn A. Seven novel Tay-Sachs mutations detected by chemical mismatch cleavage of PCR-amplified cDNA fragments. Genomics 1991; 11: 124-134.

35. Kaya N, Al-Owain M, Abudheim N, Al-Zahrani J, Colak D, Al-Sayed M, et al. GM2 gangliosidosis in Saudi Arabia: multiple mutations and considerations for future carrier screening. Am J Med Genet A 2011; 155A: 1281-1284.

36. Pomponio RJ, Coskun T, Demirkol M, Tokatli A, Ozalp I, Huner G, et al. Novel mutations cause biotinidase deficiency in Turkish children. J Inherit Metab Dis 2000; 23: 120-128.

37. Mallery DL, Tanganelli B, Colella S, Steingrimsdottir H, van Gool AJ, Troelstra C, et al. Molecular analysis of mutations in the CSB (ERCC6) gene in patients with Cockayne syndrome. Am J Hum Genet 1998; 62: 77-85.

38. Agostino A, Valletta L, Chinnery PF, Ferrari G, Carrara F, Taylor RW, et al. Mutations of ANT1, Twinkle, and POLG1 in sporadic progressive external ophthalmoplegia (PEO). Neurology 2003; 60: 1354-1356.

39. Gempel K, Topaloglu H, Talim B, Schneiderat P, Schoser BG, Hans VH, et al. The myopathic form of coenzyme Q10 deficiency is caused by mutations in the electron-transferring-flavoprotein dehydrogenase (ETFDH) gene. Brain 2007; 130: 2037-2044.

40. Tiranti V, Hoertnagel K, Carrozzo R, Galimberti C, Munaro M, Granatiero M, et al. Mutations of SURF-1 in Leigh disease associated with cytochrome c oxidase deficiency. Am J Hum Genet 1998; 63: 1609-1621.

41. Teraoka M, Yokoyama Y, Ninomiya S, Inoue C, Yamashita S, Seino Y. Two novel mutations of SURF1 in Leigh syndrome with cytochrome c oxidase deficiency. Hum Genet 1999; 105: 560-563.
